# The impact of chemotherapy-associated neutrophil/ lymphocyte counts on prognosis of adjuvant chemotherapy in colorectal cancer

**DOI:** 10.1186/1471-2407-13-177

**Published:** 2013-04-03

**Authors:** Hong Chu-Yuan, Peng Jing, Wei Yi-Sheng, Peng He-Ping, Yang Hui, Zhao Chu-Xiong, Liang Guo-Jian, Wang Guo-Qiang

**Affiliations:** 1 Department of Gastrointestinal Surgery, Lab of Surgery, the Second Affiliated Hospital of Guangzhou Medical University, 250 Chang-gang-dong Road, Guangzhou, Guangdong Province, 510260, China; 2 Department of General Surgery, Lab of Surgery, the Second Affiliated Hospital of Guangzhou Medical University, 250 Chang-gang-dong Road, Guangzhou, Guangdong Province, 510260, China; 3 Department of Gastroenterology, the Second Affiliated Hospital of Guangzhou Medical University, 250 Chang-gang-dong Road, Guangzhou, Guangdong Province, 510260, China

**Keywords:** Colorectal cancer, Chemotherapy, Lymphopenia, Neutropenia, Prognosis

## Abstract

**Background:**

Leukocytes play an important role in cancer development. However, the impact of chemotherapy-associated neutropenia/lymphopenia on the prognosis of adjuvant chemotherapy is unknown. Here, we aimed to explore the impact of chemotherapy-associated neutrophil/lymphocyte counts on prognosis of adjuvant chemotherapy in colorectal cancer (CRC) and the risk factors for developing neutropenia/lymphopenia which showed impact on the prognosis of CRC receiving adjuvant chemotherapy.

**Methods:**

From February 2003 to January 2011, 243 stage II and III CRC patients receiving adjuvant chemotherapy were enrolled in this retrospective study. The associations between neutrophil/ lymphocyte counts and disease free survival (DFS)/overall survival (OS) of CRC, and the risk factors for neutropenia/lymphopenia were investigated.

**Results:**

No association of chemotherapy-associated neutrophil counts and CRC recurrence (AUC = 0.474, P = 0.534), death (AUC = 0.449, P = 0.249) was found by ROC analysis. However, the chemotherapy-associated lymphocyte counts could significantly affect CRC recurrence (AUC = 0.634, P = 0.001), or death(AUC = 0.607, P = 0.015), with a optimized cut-off of 0.66 × 10^9^/L for recurrence, and 0.91 × 10^9^/L for death, respectively. Kaplan–Meier method showed chemotherapy-associated lymphopenia <0.66 × 10^9^/L was associated with shorter DFS (P < 0.0001), and chemotherapy-associated lymphopenia <0.91 × 10^9^/L was associated with shorter OS (P = 0.003). Cox regression model showed chemotherapy-associated lymphopenia <0.66 × 10^9^/L was the independent prognostic factor for DFS (HR, 3.521; 95%CI = 1.703-7.282), and chemotherapy-associated lymphopenia <0.91 × 10^9^/L was the independent prognostic factor for OS (HR, 2.083; 95% CI = 1.103-3.936). Multivariate logistic regression showed the risk of developing chemotherapy-associated lymphopenia <0.66 × 10^9^/L was found in those with pretreatment CEA ≥10 ng ml^-1^ (OR, 3.338; 95% CI = 1.523-7.315), and the risk of developing chemotherapy-associated lymphopenia <0.91 × 10^9^/L was found in those with age >60 years (OR, 2.872; 95% CI = 1.344-6.136).

**Conclusions:**

Chemotherapy-associated lymphopenia <0.66 × 10^9^/L /0.91 × 10^9^/L has a significant impact on the prognosis of CRC receiving adjuvant chemotherapy. Pretreatment CEA ≥10 ng ml^-1^ is the independent risk factor for developing lymphopenia <0.66 × 10^9^/L, and age >60 years is the independent risk factor for developing lymphopenia <0.91 × 10^9^/L during adjuvant chemotherapy of CRC.

## Background

Colorectal cancer (CRC) is increasing in the world and China in recent years [1-3]. 5-Fu-based chemotherapy has been used to reduce the risk of relapse after surgery. 5-Fu plus leucovorin with the addition of oxaliplatin chemotherapy(FOLFOX), which improved survival significantly compared with 5-FU alone [4], has been widely accepted as the standard adjuvant chemotherapy for stage III and stage II colorectal cancer. However, stage III patients have a 50–60% likelihood of tumor recurrence, and 20–30% of stage II patients will show recurrent disease [5]. Therefore, it is very important to select subgroups of patients who are most likely to be resistant to a given chemotherapy regimen.

In the past decades, several biomarkers such as microsatellite instability [6], Chromosome 18q allelic loss [7], TP53 mutation/overexpression [8,9], thymidylate synthase overexpression [9], Ki-67 overexpression [9], have been found to be associated with prognosis of colorectal cancer. However, some other reports failed to demonstrate the prognostic/predictive effect of the biomarkers mentioned above [10-12]. Thereby it is critical to identify the reliable biomarkers for prognosis of CRC patients receiving adjuvant chemotherapy. On the other hand, leukocytes play an important role in cancer development [13,14]. Thus, it seems that leukocytes variation may have some impact on the survival of colorectal cancer. However, whether neutropenia and lymphopenia, which are the common chemotherapy-induced toxicities, may influence the prognosis of adjuvant chemotherapy in CRC is unknown. Herein we explored the impact of chemotherapy-associated neutrophil/ lymphocyte counts on the prognosis of CRC patients receiving adjuvant chemotherapy. We also examined the risk factors affecting neutrophil or lymphocyte variation which showed impact on the prognosis of CRC patients receiving adjuvant chemotherapy to guide the individualized medicine for patients with CRC requiring chemotherapy.

## Methods

### Patient selection

From February 2003 to January 2011, stage II and III pathology-proven CRC patients who received FOLFOX regimen as adjuvant chemotherapy in the Second Affiliated Hospital of Guangzhou Medical University were enrolled in our retrospective study. Other eligibility criteria were as follows: At least 3 cycles of adjuvant chemotherapy, no tumor recurrence during chemotherapy, WHO performance status (PS) 0–1, adequate pretreatment renal (pretreatment creatinine clearance ≥60 mL/min), and hepatic functions (pretreatment bilirubin ≤1.5 upper limit of normal, pretreatment alanine aminotransferase and/or aspartate aminotransferase ≤2.5 upper limit of normal), adequate baseline bone marrow (absolute baseline neutrophil counts ≥ 2.0 × 10^9^ cells/L, absolute baseline lymphocyte counts ≥1.0 × 10^9^ cells/L, baseline platelet counts ≥100 × 10^9^ cells/L). The exclusion criteria included the following: biologic or immunotherapy, concomitant or neoadjuvant radiotherapy, previous systemic chemotherapy or neoadjuvant chemotherapy, primary prophylactic administration of granulocyte colony-stimulating factor (G-CSF) following chemotherapy, previous malignancies other than colorectal cancer, documented human immunosuppression. The evaluation of WHO PS and blood cell counts were performed before each next chemotherapy cycle and the lowest blood cell count was recorded in our study. The study was approved by the institutional review boards of Guangzhou Medical University.

### FOLFOX Treatment

The FOLFOX regimen consisted of a 2-h intravenous infusion of oxaliplatin (85 mg/m^2^) and folinic acid (400 mg/m^2^), followed by an intravenous bolus injection of 5-FU (400 mg/m^2^) plus a 46-h intravenous infusion of 5-FU (2400 mg/m^2^), repeated every 2 weeks. Chemotherapy was delayed due to severe toxicity and the doses of oxaliplatin and 5-FU were reduced by 15% in subsequent cycles. Chemotherapy was discontinued due to unacceptable toxicity.

### Data collection and assessment of adjuvant chemotherapy prognosis

From the medical records we collected the data of pretreatment albumin, pretreatment carcinoembryonic antigen (CEA), differentiation, sex, age, location, stage, blood cell counts. Albumin was divided into the following two groups: ≥ or < 35 g/L. CEA was divided into the following two groups: ≥ or < 10 ng ml^-1^. Duration of neutropenia <1.5 × 10^9^/L / lymphopenia <1.0 × 10^9^ L was divided into the following two groups: > 28 days or ≤28 days, respectively. Staging was performed according to the American Joint Committee on Cancer (AJCC, seventh edition).

### Statistical analysis

The prognostic value of chemotherapy-associated neutrophil/lymphocyte counts on tumor recurrence, death was analyzed by Receiver Operating Characteristic (ROC) analysis to select the best cut-off of neutrophil/lymphocyte counts. Kaplan–Meier method was used to estimate disease free survival (DFS) / overall survival (OS). Multivariate analysis and Cox proportional hazard models were used to determine the independent impact of chemotherapy-associated neutrophil/lymphocyte counts on DFS/OS. The risk factors for neutrophil or lymphocyte counts abnormality which showed impact on DFS/OS of stage II-III CRC receiving adjuvant chemotherapy were analyzed by the unconditional logistic regression model. All statistical analyses were performed by Statistical Package of Social Sciences 17.0 software and 2-sided. P value <0.05 was considered to be statistically significant.

## Results

### Characteristics of the study population

There were 243 CRC patients who fulfilled the eligibility and exclusion criteria in the present study. The characteristics of the studied population were shown in Table 1. Of the 243 cases, 21 cases had to stop adjuvant chemotherapy for severe toxicity. The median age of 243 cases was 59 years with a range of 22–82 years. The median follow-up time was 31 months (range 7–103 months). Of the 243 cases, 109(44.9%) were female and 134(55.1%) were male. 111(45.7%) cases were rectal cancer, 77(31.7%) cases were left colon cancer, and 55 (22.6%) were right colon cancer. According to the American Joint Committee on Cancer (AJCC, seventh edition), there were 140(57.6%) stage II cases and 103(42.4%) stage III cases. The population in our study had a mean baseline neutrophil counts of 4.27 ± 1.49 × 10^9^/L, a mean baseline lymphocyte counts of 1.88 ± 0.59 × 10^9^/L. 90(37.0%) cases experienced neutropenia <1.5 × 10^9^/L and 104(42.8%) cases experienced lymphopenia <1.0 × 10^9^/L during chemotherapy. According to National Cancer Institute Common Terminology Criteria for Adverse Events version 3.0 (NCI-CTC), grade 3/4 neutropenia was observed in 49(20.2%) cases and grade 3/4 lymphopenia was observed in 15(6.2%) cases. 4(1.6%) cases suffered from neutropenic infection requiring hospitalization and treatment with intravenous antibiotics at 5 cycles of chemotherapy.

**Table 1:** Patient characteristics

**Variables**	**Number of cases**	**Percentage(%)**
**Follow-up(months)**		
Median(range)	31(7–103)	
**Age(years)**		
Median(range)	59(22–82)	
**Pretreatment albumin**		
≥35 g/L	173	71.2
<35 g/L	70	28.8
**Pretreatment CEA**^**a**^		
≤10 ng/ml	186	76.5
>10 ng/ml	57	23.5
**Differentiation**		
High	24	9.9
Middle	198	81.5
Low	21	8.6
**Sex**		
Male	134	55.1
Female	109	44.9
**Age**		
≤49 years	56	23.0
50-60 yeas	72	29.6
>60 years	115	47.3
**am**		
Rectum	111	45.7
Left colon cancer	77	31.7
Right colon cancer	55	22.6
**Stage**		
II	140	57.6
III	103	42.4
**Baseline neutrophil counts(×10**^**9**^**/L)**		
Mean(± SD)	4.27 ± 1.49	
**Baseline lymphocyte counts(×10**^**9**^**/L)**		
Mean(± SD)	1.88 ± 0.59	
**Chemotherapy-associated neutrophil counts**		
Neutrophil counts ≥ 1.5 × 10^9^/L	153	63.0
Neutropenia < 1.5 × 10^9^/L	90	37.0
**Chemotherapy-associated lymphocyte counts**		
Lymphocyte counts ≥ 1.0 × 10^9^/L	139	57.2
Lymphopenia < 1.0 × 10^9^/L	104	42.8

^a^ Carcinoembryonic antigen.

### The prognostic value of chemotherapy-associated neutrophil/ lymphocyte counts on CRC recurrence/death

**Figure 1:**
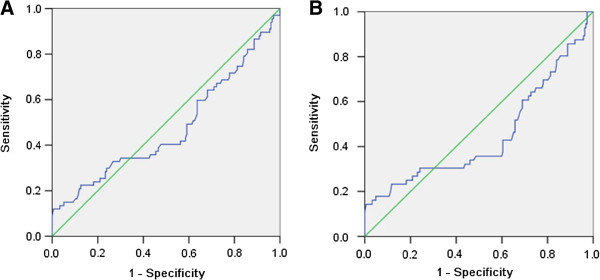
**ROC curve of chemotherapy-associated neutrophil counts affecting CRC recurrence, death.** (**A**) The area under curve (AUC) of chemotherapy-associated neutrophil counts for CRC recurrence was 0.474 and P value was 0.534. (**B**) The AUC of chemotherapy-associated neutrophil counts for CRC death was was0.449 and P value was 0.249.

**Figure 2:**
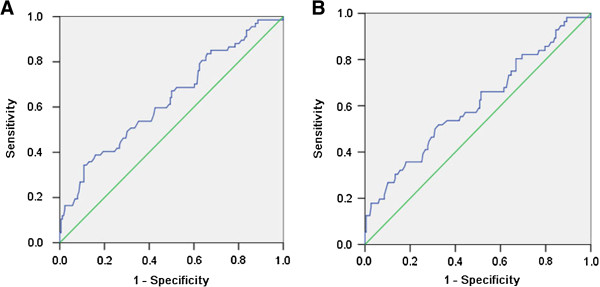
**ROC curve of chemotherapy-associated lymphocyte counts affecting CRC recurrence, death.** (**A**) The area under curve (AUC) of chemotherapy-associated neutrophil counts for CRC recurrence was 0.634 and P value was 0.001. (**B**) The AUC of chemotherapy-associated neutrophil counts for CRC death was was 0.607 and P value was 0.015.

The cut-offs according to NCI-CTC may not be reliable to evaluate the impact of chemotherapy-associated neutrophil/lymphocyte counts on the prognosis of CRC receiving adjuvant chemotherapy. We investigated the prognostic value of chemotherapy-associated neutrophil/lymphocyte counts on CRC recurrence, death by ROC analysis to select the best cut-off. No association of chemotherapy-associated neutrophil counts and tumor recurrence, death was found. The area under curve (AUC) of chemotherapy-associated neutrophil counts for CRC recurrence, death was 0.474(P = 0.534), 0.449(P = 0.249), respectively (Figure 1). However, the significance was found between chemotherapy-associated lymphocyte counts and CRC recurrence, death. The AUC of chemotherapy-associated lymphocyte counts, was 0.634 for CRC recurrence (P = 0.001), 0.607 for death (P = 0.015), respectively (Figure 2). We then evaluated the best cut-off of chemotherapy-associated lymphocyte level affecting CRC recurrence, death with the highest value of sensitivity plus specificity by ROC analysis. The best prognostic cut-off was 0.66 × 10^9^/L for CRC recurrence, 0.91 × 10^9^/L for death, respectively. 42(17.3%) cases had chemotherapy-associated lymphopenia < 0.66 × 10^9^/L and 90(37.0%) cases had chemotherapy-associated lymphopenia < 0.91 × 10^9^/L in this study.

### The prognostic value of chemotherapy-associated lymphopenia <0.66 × 10^9^/L, 0.91 × 10^9^/L for colorectal cancer

**Figure 3:**
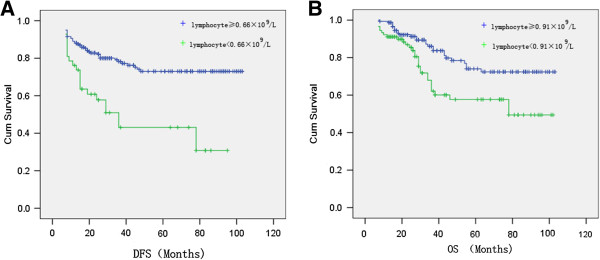
**DFS, OS curve by Kaplan–Meier method.** (**A**) Chemotherapy-associated lymphopenia <0.66 × 10^9^/L was associated with shorter DFS and P value was <0.0001. (**B**) Chemotherapy-associated lymphopenia <0.91 × 10^9^/L was associated with shorter OS and P value was 0.003.

Therefore, we selected 0.66 × 10^9^/L, 0.91 × 10^9^/L which were found by ROC analysis as the cut-offs to investigate the impact of chemotherapy-associated lymphopenia on DFS, OS of CRC receiving adjuvant chemotherapy. Kaplan–Meier method showed chemotherapy-associated lymphopenia <0.66 × 10^9^/L was associated with shorter DFS (P < 0.0001), whereas chemotherapy-associated lymphopenia <0.91 × 10^9^/L was associated with shorter OS (P = 0.003) (Figure 3). To control the possible confounding of the main effects of chemotherapy-associated lymphopenia <0.66 × 10^9^/L/0.91 × 10^9^/L on DFS/OS, respectively, the clinicopathological factors (pretreatment albumin, pretreatment CEA, differentiation, sex, age, location and stage) and duration of lymphopenia <1.0 × 10^9^ L were further adjusted for in the multivariate cox regression model. As shown in Table 2, cox regression model showed chemotherapy-associated lymphopenia <0.66 × 10^9^/L (HR, 3.521; 95% CI = 1.703-7.282), pretreatment CEA ≥10 ng ml^-1^ (HR, 1.827; 95% CI = 1.040-3.211), stage III (HR, 2.723; 95% CI = 1.549-4.786) were independent prognostic factors for DFS, and 99 (40.7%) cases had all of those negative prognostic factors. Moreover, as shown in Table 3, cox regression model showed chemotherapy-associated lymphopenia <0.91 × 10^9^/L (HR, 2.083 ; 95% CI = 1.103-3.936), pretreatment CEA ≥10 ng ml^-1^ (HR, 1.900; 95% CI = 1.056-3.416), stage III (HR, 3.641; 95% CI = 1.980-6.697) were independent prognostic factors for OS, and 77 (31.7%) cases had all of those negative prognostic factors.

**Table 2:** **Prognostic value of chemotherapy-associated lymphopenia <0.66 × 10**^**9**^**/L for DFS of colorectal cancer**

**Variables**	**Hazard ratio**	**95% confidence intervals**	**P-value**
**Pretreatment albumin**			
≥35 g/L	1.00(ref.)		
<35 g/L	1.000	0. 592–1. 692	0.999
**Pretreatment CEA**^**a**^			
<10 ng ml^-1^	1.00(ref.)		
≥10 ng ml^-1^	1. 827	1.040-3.211	0.036
**Differentiation**			
Well	1.00(ref.)		
Moderately	0. 676	0. 292–1.567	0.361
Low	0.882	0.294-2.644	0.823
**Sex**			
Male	1.00(ref.)		
Female	1.018	0.616-1.682	0.946
**Age**			
≤49 years	1.00(ref.)		
50-60 yeas	1.612	0.750-3.465	0.222
>60 years	1.386	0.673-2.854	0.376
**Location**			
Rectum	1.00(ref.)		
Left colon cancer	0.873	0.483-1.576	0.651
Right colon cancer	0.850	0.438-1.649	0.630
**Stage**			
II	1.00(ref.)		
III	2.723	1.549-4.786	0.001
**Duration of lymphopenia <1.0 × 10**^**9**^ **L**			
≤28 days	1.00(ref.)		
> 28 days	0.556	0.243-1.273	0.165
**Chemotherapy-associated lymphocyte counts**			
Lymphocyte counts ≥ 0.66 × 10^9^/L	1.00(ref.)		
Lymphopenia <0.66 × 10^9^/L	3.521	1.703-7.282	0.001

^a^ Carcinoembryonic antigen.

**Table 3:** **Prognostic value of chemotherapy-associated lymphopenia <0.91 × 10**^**9**^**/L for OS of colorectal cancer**

**Variables**	**Hazard ratio**	**95% confidence intervals**	**P-value**
**Pretreatment albumin**			
≥35 g/L	1.00(ref.)		
<35 g/L	1.140	0.648-2.007	0.649
**Pretreatment CEA**^**a**^			
<10 ng ml^-1^	1.00(ref.)		
≥10 ng ml^-1^	1. 900	1.056-3.416	0.032
**Differentiation**			
Well	1.00(ref.)		
Moderately	0. 865	0. 323–2.314	0.772
Low	1.097	0.311-3.871	0.885
**Sex**			
Male	1.00(ref.)		
Female	0.856	0.493-1.489	0.583
**Age**			
≤49 years	1.00(ref.)		
50-60 yeas	1.514	0.625-3.666	0.359
>60 years	1.587	0.698-3.607	0.271
**Location**			
Rectum	1.00(ref.)		
Left colon cancer	1.067	0.564-2.019	0.843
Right colon cancer	0.647	0.296-1.414	0.275
**Stage**			
II	1.00(ref.)		
III	3.641	1.980-6.697	<0.0001
**Duration of lymphopenia <1.0 × 10**^**9**^ **L**			
≤28 days	1.00(ref.)		
> 28 days	0.770	0.341-1.740	0.530
**Chemotherapy-associated lymphocyte counts**			
Lymphocyte counts ≥ 0.66 × 10^9^/L	1.00(ref.)		
Lymphopenia <0.66 × 10^9^/L	2.083	1.103-3.936	0.024

^a^ Carcinoembryonic antigen.

### The risk factors for chemotherapy-associated lymphopenia <0.66×10^9^/L /0.91 × 10^9^/L in colorectal cancer

Then the clinicopathological factors (pretreatment albumin, pretreatment CEA, differentiation, sex, age, location, stage) were included in the multivariate logistic regression model to explore the independent risk factors for chemotherapy-associated lymphopenia <0.66 × 10^9^/L**/**0.91 × 10^9^/L in colorectal cancer. As shown in Table 4 and Table 5, pretreatment CEA ≥10 ng ml^-1^ was the independent risk factor for chemotherapy-associated lymphopenia <0.66 × 10^9^/L(OR, 3.338; 95% CI = 1.523-7.315), and age >60 years was the independent risk factor for chemotherapy-associated lymphopenia <0.91 × 10^9^/L(OR, 2.872; 95% CI = 1.344-6.136).

**Table 4:** **The risk factors for chemotherapy-associated lymphopenia <0.66 × 10**^**9**^**/L in colorectal cancer by multivariate logistic regression model**

**Variables**	**Odds ratio**	**95% confidence intervals**	**P-value**
**Pretreatment albumin**			
≥35 g/L	1.00(ref.)		
<35 g/L	0.685	0.302-1.550	0.363
**Pretreatment CEA**^**a**^			
<10 ng ml^-1^	1.00(ref.)		
≥10 ng ml^-1^	3.338	1.523-7.315	0.003
**Differentiation**			
Well	1.00(ref.)		
Moderately	1.200	0.308-4.678	0.793
Low	3.049	0.553-16.828	0.201
**Sex**			
Male	1.00(ref.)		
Female	1.219	0.600-2.474	0.584
**Age**			
≤49 years	1.00(ref.)		
50-60 yeas	0.840	0.271-2.608	0.763
>60 years	2.358	0.904-6.154	0.080
**Location**			
Rectum	1.00(ref.)		
Left colon cancer	1.056	0.484-2.300	0.892
Right colon cancer	0.532	0.192-1.475	0.225
**Stage**			
II	1.00(ref.)		
III	0.847	0.403-1.781	0.661

^a^ Carcinoembryonic antigen.

**Table 5:** **The risk factors for chemotherapy-associated lymphopenia <0.91 × 10**^**9**^**/L in colorectal cancer by multivariate logistic regression model**

**Variables**	**Odds ratio**	**95% confidence intervals**	**P-value**
**Pretreatment albumin**			
≥35 g/L	1.00(ref.)		
<35 g/L	1.410	0.772-2.576	0.263
**Pretreatment CEA**^**a**^			
<10 ng ml^-1^	1.00(ref.)		
≥10 ng ml^-1^	1.885	0.984-3.610	0.056
**Differentiation**			
Well	1.00(ref.)		
Moderately	1.590	0.564-4.483	0.381
Low	2.694	0.694-10.458	0.152
**Sex**			
Male	1.00(ref.)		
Female	1.725	0.987-3.016	0.056
**Age**			
≤49 years	1.00(ref.)		
50-60 yeas	1.617	0.705-3.709	0.256
>60 years	2.872	1.344-6.136	0.006
**Location**			
Rectum	1.00(ref.)		
Left colon cancer	0.948	0.502-1.789	0.869
Right colon cancer	0.780	0.381-1.596	0.496
**Stage**			
II	1.00(ref.)		
III	0.881	0.494-1.572	0.668

^a^ Carcinoembryonic antigen.

## Discussion

In the present study, we investigated the impact of chemotherapy-associated neutrophil/lymphocyte counts on prognosis of CRC receiving adjuvant chemotherapy. We found chemotherapy-associated lymphopenia, but not neutropenia to be associated with CRC recurrence, death after adjuvant chemotherapy. The best lymphopenic cut-off affecting CRC recurrence was 0.66 × 10^9^/L, and that affecting CRC death was 0.91 × 10^9^/L. Chemotherapy-associated lymphopenia <0.66 × 10^9^/L/0.91 × 10^9^/L was the independent prognostic factor for worse DFS/OS, respectively, in stage II and III CRC receiving adjuvant chemotherapy. Pretreatment CEA ≥10 ng ml^-1^ was the independent risk factor for chemotherapy-associated lymphopenia <0.66 × 10^9^/L, and age >60 years was the independent risk factor for chemotherapy-associated lymphopenia <0.91 × 10^9^/L in CRC receiving adjuvant chemotherapy.

It is reported that circulating lymphocytes play a central role in anti-tumor effect. Lymphodepletion due to the altered bone marrow microenvironment was observed in the ApcMin/+ mouse model of intestinal tumorigenesis [15], which indicates the lymphodepletion may contribute to the drop of anti-tumor immune and tumorigenesis. Due to interplay between soluble factors such as cytokines, chemokines, surface receptors and adhesion molecules, progression and invasion occur in a dynamic microenvironment involving the complex communications between tumor cells and many types of immune cells including the various types of lymphocytes [16]. Thus,lymphopenia is supposed to account for the adverse anti-tumor microenvironment. Moreover, lymphopenia is associated with increased circulating levels of IL-7 [17,18]. IL-7 plays an oncogenic role promoting proliferation, lymphangiogenesis and metastasis in neoplasm [19-23]. That may partly explain the clinical outcome of chemotherapy-associated lymphopenia in colorectal cancer in our study. Recently, pretreatment peripheral blood lymphocytes have been found to show a significant impact on the complete response rate in response to preoperative radiotherapy in locally advanced rectal cancer (RC) patients and lymphocyte-mediated immune reactions are supposed to have positive roles in radiosensitivity for RC [24]. Likewise, pretreatment lymphopenia is an independent risk factor for shorter survival of palliative chemotherapy in colorectal cancer [25]. Moreover, pretreatment lymphopenia has been found to be the independent risk factor for Peripheral T-cell lymphoma, not otherwise specified (PTCL-NOS) [26], metastatic breast carcinoma [27], advanced soft tissue sarcoma [27] and non-Hodgkin's lymphoma [27]. Lymphopenia is a common adverse event during chemotherapy with the incidence >25% [28,29]. However, the clinical significance of chemotherapy-associated lymphocyte drop in the adjuvant chemotherapy of CRC is unclear. That indicated lymphopenia, which is the common chemotherapy-induced toxity, may be the possible mechanism for the failure of adjuvant chemotherapy in CRC. Our study also showed chemotherapy-associated neutropenia had no impact on DFS, OS of CRC, suggesting lymphopenia rather than neutropenia may play an important role in the variation of anti-tumor immune reaction during adjuvant chemotherapy in CRC. Chemotherapy-associated lymphopenia is reported rarely in clinical trials. To the best of our knowledge, our study demonstrated the impact of chemotherapy-associated lymphopenia on the clinical outcome of adjuvant chemotherapy in CRC for the first time.

The cut-off of lymphopenia is different in previous studies. Some reports defined lymphopenia as a lymphocyte count of less than 1.5 × 10^9^/L [30-32], while some other reports chose the threshold level of 1.0 × 10^9^/L [25,27]. However, when we chose the threshold level of 1.0 × 10^9^/L in the present study, lymphopenia <1.0 × 10^9^/L was not the independent risk factor for worse DFS,OS in stage II and III CRC receiving adjuvant chemotherapy(data not shown). Therefore, the cut-offs mentioned above may not be suitable for the evaluation of lymphocyte counts on prognosis of CRC receiving adjuvant chemotherapy. ROC analysis showed the best cut-off of lymphopenia was 0.66 × 10^9^/L for CRC recurrence, <0.91 × 10^9^/L for CRC death in this study and Cox regression model as well as Kaplan–Meier method confirmed the prognostic value of lymphopenia <0.66 × 10^9^/L for DFS,lymphopenia <0.91 × 10^9^/L for OS. Thus, it is reasonable that chemotherapy-associated lymphopenia <0.66 × 10^9^/L/0.91 × 10^9^/L is a simple biomarker affecting worse DFS/OS,respectively, for stage II and III CRC receiving adjuvant chemotherapy. Lymphopenia <0.66 × 10^9^/L/0.91 × 10^9^/L can be the cut-offs to guide the individualized medicine in adjuvant chemotherapy of colorectal cancer.

Moreover, though chemotherapy associated lymphopenia <0.66 × 10^9^/L/0.91 × 10^9^/L showed a longer duration of duration of lymphopenia <1.0 × 10^9^ L compared with lymphopenia ≥0.66 × 10^9^/L/0.91 × 10^9^/L (80.76 ± 49.64 days vs 7.43 ± 12.38 days, 51.84 ± 43.79 days vs 1.43 ± 5.01 days, respectively, data not shown), cox regression model showed lymphopenia <0.66 × 10^9^/L/0.91 × 10^9^/L, but not duration of lymphopenia <1.0 × 10^9^ L, was the independent prognostic factor. That suggest chemotherapy-associated lymphopenia level, rather than duration of lymphopenia <1.0 × 10^9^/L, may play an important role in the prognosis of CRC receiving adjuvant chemotherapy.

The previous studies reported lymphopenia could be affected by various factors including vitamin D deficiency [32], weight loss [33], variation of circulating metal ions levels [34], malnutrition [35]. However, our study showed pretreatment CEA ≥10 ng ml^-1^ was the only independent risk factor for chemotherapy-associated lymphopenia <0.66 × 10^9^/L, and age >60 years was the only independent risk factor for chemotherapy-associated lymphopenia <0.91 × 10^9^/L in colorectal cancer, suggesting those who have a pretreatment CEA ≥10 ng ml^-1^ are predispose to lymphopenia <0.66 × 10^9^/L, meanwhile those >60 years old are predispose to lymphopenia <0.91 × 10^9^/L during adjuvant chemotherapy. Thus, pretreatment CEA ≥10 ng ml^-1^ and age > 60 years should be taken into account in the individualized medicine in adjuvant chemotherapy of CRC to reduce the risk of chemotherapy-associated lymphopenia and improve survival. The rate of grade 3/4 neutropenia in our study was lower than that in the MOSAIC study (41.1%) [36]. In MOSAIC study, FOLFOX was administered to those who have neutrophil counts >1.5 × 109/L, while those with baseline neutrophil counts ≥ 2.0 × 10^9^ cells/L were included in our study. That may contribute to the difference in the severity of neutropenia.

However, there are some limitations in the present study. The sample size was relatively small. Therefore the conclusions, such as the cut-offs of lymphopenia, should be proven by the larger, multicenter study. We also await the further functional analyses of circulating lymphocyte subpopulations that contribute to prognosis of CRC receiving adjuvant chemotherapy.

## Conclusions

In conclusion, our study indicated chemotherapy-associated lymphopenia <0.66 × 10^9^/L/0.91 × 10^9^/L was associated with worse prognosis in adjuvant chemotherapy of CRC. Chemotherapy-associated lymphopenia is supposed to reflect the immunosuppression state which has an adverse impact on the anti-tumor effect and may partly explain the failure of adjuvant chemotherapy in CRC. Circulating lymphocyte counts should be monitored during chemotherapy to guide individualized medicine in adjuvant chemotherapy of CRC, especially for those who have a pretreatment CEA ≥10 ng ml^-1^ or those >60 years old.

## Abbreviations

CRC: Colorectal cancer; FOLFOX: 5-Fu plus leucovorin with the addition of oxaliplatin chemotherapy; DFS: Disease free survival; PS: Performance status; G-CSF: Granulocyte colony-stimulating factor; CEA: Carcinoembryonic antigen; OS: Overall survival; ROC: Receiver Operating Characteristic; AJCC: The American Joint Committee on Cancer; AUC: Area under curve; RC: Rectal cancer

## Competing interests

The authors declare that they have no competing interests.

## Authors’ contributions

HCY and WYS designed the study, performed the statistical analysis and drafted the manuscript. PJ and WGQ collected the clinical data. PHP, YH, ZCX, LGJ participated in its design and coordination and helped to draft the manuscript. All authors read and approved the final manuscript.

## Pre-publication history

The pre-publication history for this paper can be accessed here:

http://www.biomedcentral.com/1471-2407/13/177/prepub
